# Establishing diagnostic thresholds for Alzheimer's disease in adults with Down syndrome: the Cambridge Examination for Mental Disorders of Older People with Down's Syndrome and Others with Intellectual Disabilities (CAMDEX-DS)

**DOI:** 10.1192/bjo.2021.36

**Published:** 2021-04-13

**Authors:** Jessica A. Beresford-Webb, Elijah Mak, Monika Grigorova, Samuel J. Daffern, Anthony J. Holland, Shahid H. Zaman

**Affiliations:** Department of Psychiatry, University of Cambridge, UK; Department of Psychiatry, University of Cambridge, UK; Department of Psychiatry, University of Cambridge, UK; Department of Genetics, University of Cambridge, UK; Department of Psychiatry, University of Cambridge, UK; Department of Psychiatry, University of Cambridge, UK

**Keywords:** Dementia, intellectual disability, imaging, CAMDEX-DS, Alzheimer's disease

## Abstract

**Background:**

Diagnosis of prodromal Alzheimer's disease and Alzheimer's disease dementia in people with Down syndrome is a major challenge. The Cambridge Examination for Mental Disorders of Older People with Down's Syndrome and Others with Intellectual Disabilities (CAMDEX-DS) has been validated for diagnosing prodromal Alzheimer's disease and Alzheimer's disease dementia, but the diagnostic process lacks guidance.

**Aims:**

To derive CAMDEX-DS informant interview threshold scores to enable accurate diagnosis of prodromal Alzheimer's disease and Alzheimer's disease dementia in adults with Down syndrome.

**Method:**

Psychiatrists classified participants with Down syndrome into no dementia, prodromal Alzheimer's disease and Alzheimer's disease dementia groups. Receiver operating characteristic analyses assessed the diagnostic accuracy of CAMDEX-DS informant interview-derived scores. Spearman partial correlations investigated associations between CAMDEX-DS scores, regional Aβ binding (positron emission tomography) and regional cortical thickness (magnetic resonance imaging).

**Results:**

Diagnostic performance of CAMDEX-DS total scores were high for Alzheimer's disease dementia (area under the curve (AUC), 0.998; 95% CI 0.953–0.999) and prodromal Alzheimer's disease (AUC = 0.954; 95% CI 0.887–0.982) when compared with healthy adults with Down syndrome. When compared with those with mental health conditions but no Alzheimer's disease, CAMDEX-DS Section B scores, denoting memory and orientation ability, accurately diagnosed Alzheimer's disease dementia (AUC = 0.958; 95% CI 0.892–0.984), but were unable to diagnose prodromal Alzheimer's disease. CAMDEX-DS total scores exhibited moderate correlations with cortical Aβ (*r* ~ 0.4 to 0.6, *P* ≤ 0.05) and thickness (*r* ~ −0.4 to −0.44, *P* ≤ 0.05) in specific regions.

**Conclusions:**

CAMDEX-DS total score accurately diagnoses Alzheimer's disease dementia and prodromal Alzheimer's disease in healthy adults with Down syndrome.

Adults with Down syndrome have an increased incidence of Alzheimer's disease,^1^ with an average age at dementia diagnosis of 55 years.^2^ The increased risk for Alzheimer's disease results from the triplication of the *amyloid precursor protein* gene on chromosome 21, leading to brain pathology indicative of Alzheimer's disease, including the deposition of amyloid-β (Aβ) plaques and cerebral atrophy.^3^ The increased prevalence and early onset of Alzheimer's disease in adults with Down syndrome highlights the need for accurate diagnosis of dementia to ensure those affected receive appropriate support, and that clinical trials aimed at preventing or delaying Alzheimer's disease in this population can be undertaken. To facilitate these efforts, diagnostic instruments that are easy to use at a single time point, do not require repeat measures and have clear standardised diagnostic guidance are required. Diagnosing dementia in people with Down syndrome is challenging because of the distinctive progression of Alzheimer's disease in this population, the presence of pre-existing cognitive deficits and an uncertain cognitive and functional baseline owing to varying levels of intellectual disability. First published in 2006, the Cambridge Examination for Mental Disorders of Older People with Down's Syndrome and Others with Intellectual Disabilities (CAMDEX-DS) informant interview is a diagnostic instrument carried out with a caregiver, and focuses on an individual's decline from their best level of functioning.^4^ Designed specifically for use in the Down syndrome population and based on standardised international criteria, the CAMDEX-DS is widely used in studies of Alzheimer's disease in people with Down syndrome. However, despite the reliability and validity observed for the CAMDEX-DS, there is no formula or threshold score that denotes an explicit diagnosis.^4^ Although the CAMDEX-DS does provide guidance regarding the necessary features required for a clinical diagnosis, a CAMDEX-DS diagnosis ultimately relies upon clinical judgement; specialist knowledge and clinical experience are essential. This study aims to codify CAMDEX-DS scores to determine whether cut-off scores can be derived that enable a better standardisation of dementia diagnosis in adults with Down syndrome. To explore the biological validity of the CAMDEX-DS scores, we hypothesised that differences in scores would be associated with variations in cortical thickness and Aβ binding in the brain, and would predict follow-up diagnoses.

## Method

### Participants

This study was part of a larger and comprehensive study of dementia in people with Down syndrome. Participants for these studies were identified in England and Scotland, via the Down's Syndrome Association or from responses to a participant call placed on our study group website. The authors assert that all procedures contributing to this work comply with the ethical standards of the relevant national and institutional committees on human experimentation and with the Helsinki Declaration of 1975, as revised in 2008. All procedures involving human patients were approved by the National Research Ethics Committee of East of England (approval numbers 11/EE/0348 and 18/EE/1118) and the Queen Square National Research Ethics Service (approval number 14/LO/1411), and the positron emission tomography (PET) brain scans were approved by the Administration of Radioactive Substances Advisory Committee. Written informed consent was obtained from all participants with Down syndrome who had the capacity to consent. For those without capacity to consent, the procedures specified in the England and Wales Mental Capacity Act (2005) or the Adults with Incapacity (Scotland) Act 2000 were adhered to.

### Clinical assessment and scoring

The CAMDEX-DS was carried out with caregivers who had known the person with Down syndrome for a minimum of 6 months. The CAMDEX-DS is a structured health interview validated for the detection of dementia in people with Down syndrome and other intellectual disabilities, and comprises four parts: Part 1, patient's/participant's best level of functioning; Part 2, cognitive and functional decline; Part 3, mental health and Part 4, physical health.^4^ For the purposes of establishing diagnostic threshold scores, we focused on Part 2, as this information is the most pertinent regarding the diagnosis. Part 2 comprises 54 questions that record changes in functions known to decline with dementia (for a list of questions included for scoring, see Supplementary Appendix 1 available at https://doi.org/10.1192/bjo.2021.36), within four subsections: Section A, everyday skills; Section B, memory and orientation; Section C1, other cognitive skills and Section C2, personality, behaviour and self-care. These questions were codified as follows: no deterioration scored nil points, slight deterioration scored one point and great deterioration scored two points. Following this scoring system, a maximum total score of 108 was established, comprising the following subsection scores: 14 for Section A, 22 for Section B, 30 for Section C1 and 42 for Section C2. A high score is therefore indicative of decline.

### Diagnostic categories

An experienced clinician (A.J.H. or S.H.Z.), blinded to the age of the participant, reviewed CAMDEX-DS interviews and, using the ICD-10 diagnostic criteria specified within the CAMDEX-DS, classified participants into the following diagnostic groups: asymptomatic (DSasymptomatic), when there was no clinical suspicion of Alzheimer's disease or evidence of a mental health condition; mental health positive (DSmentalhealth+), when there was no clinical suspicion of Alzheimer's disease but there was evidence of a mental health condition; prodromal Alzheimer's disease (DSprodromal), when there was a suspicion of Alzheimer's disease but symptoms did not fulfil all the criteria for dementia; and Alzheimer's disease dementia (DSdementia), when criteria are met for Alzheimer's disease dementia. Participants without a diagnosis of prodromal Alzheimer's disease or Alzheimer's disease were divided into either the DSasymptomatic or DSmentalhealth+ diagnostic group because of the potential for any existing mental health conditions (identified from Part 3 of the CAMDEX-DS interview schedule) to have a cognitive and functional effect, and thus increase the CAMDEX-DS score. The mental health conditions screened for were depression, anxiety, paranoid illness and clouding/delirium, as set out in Part 3 of the CAMDEX-DS interview. No participant in this study had a history of substance misuse. The diagnostic groups DSprodromal and DSdementia included individuals who exhibited symptoms associated with the mental health conditions screened for (see above). However, the reported cognitive and functional change (identified in Part 2 of the CAMDEX-DS interview) was considered to be best explained by the beginnings of dementia or dementia itself.

### Structural magnetic resonance imaging and carbon-11-labeled Pittsburgh compound B PET imaging

Magnetic resonance imaging (MRI) and PET neuroimaging was completed in a subset of participants. PET scans were acquired in three-dimensional mode, on a General Electric Medical Systems Advanced PET Scanner, using carbon-11-labeled Pittsburgh compound B. Aβ load was calculated in all cortical regions, using the nondisplaceable binding potential (BP_ND_). MRI scans were completed on a 3-Tesla Siemens Magnetom Verio Scanner (Siemens AG, Germany). Cortical thickness was assessed with FreeSurfer (Mac OS X, version 5.3; see http://surfer.nmr.mgh.harvard.edu/), using the protocol devised by Fischl and Dale.^5^ Full details on the imaging data acquisition are published in Annus et al.^6^

### Statistical analysis

Statistical analysis was completed with SPSS software package (Mac OS X, version 26.0), and the *R* statistical package (Mac OS X, version 1.2.5033). Kruskal–Wallis tests evaluated differences in CAMDEX-DS scores between the DSasymptomatic, DSmentalhealth+, DSprodromal and DSdementia groups. A Dunn's test with Bonferroni correction assessed the significance. *ε*^2^ was used as an effect size to indicate the magnitude of the difference between groups.^7^ Receiver operating characteristic (ROC) analyses were performed to evaluate the ability of CAMDEX-DS scores to diagnose prodromal Alzheimer's disease and Alzheimer's disease dementia. The Youden Index for each potential CAMDEX-DS score value was calculated as the sensitivity plus the specificity minus one, and the value with the maximum Youden Index was selected as the cut-off value. For high areas under the curve (AUCs) (>0.90), the Wilson score interval was used to calculate confidence intervals. Spearman partial correlations tested associations between CAMDEX-DS total score and regional BP_ND_, and CAMDEX-DS total score and regional cortical thickness (measured in millimetres), adjusted for age and level of intellectual disability. *P*-values were adjusted with the Bonferroni method set at *P* ≤ 0.05.

## Results

### Participants

CAMDEX-DS interviews were obtained from 85 participants (see Table 1, age range 19–65 years, 42 women, 33 mild intellectual disability, 48 moderate intellectual disability). A total of 11 were classified as DSdementia, 10 as DSprodromal, 15 as DSmentalhealth+ and 49 as DSasymptomatic. Kruskal–Wallis tests reported demographic differences between the diagnostic groups (age: *χ*^2^(3) = 26.422, *P* < 0.001, *ε*^2^ = 0.315; gender: *χ*^2^(3) = 9.173, *P* = 0.027, *ε*^2^ = 0.109). *Post-hoc* Dunn's tests with Bonferroni correction found that the age and gender of participants in the DSmentalhealth+ and DSasymptomatic groups did not differ significantly (age, *P* = 1; gender, *P* = 1). Those in the DSdementia and DSprodromal groups were older than those in the DSasymptomatic group (DSdementia versus DSasymptomatic, *P* < 0.001; DSprodromal versus DSasymptomatic, *P* = 0.010) and those in the DSmentalhealth+ group (DSdementia versus DSmentalhealth+, *P* = 0.001; DSprodromal versus DSmentalhealth+, *P* = 0.010). Of the total 85 participants, 39 underwent amyloid PET and structural MRI scans (Supplementary Table 1).

**Table 1:** Participant characteristics within each diagnostic group

Whole sample	DSasymptomatic	DSmentalhealth+	DSprodromal	DSdementia
Number of participants	49	15	10	11
Age at CAMDEX-DS interview, years, mean (s.d.)	37.35 (9.30)	35.93 (6.88)	47.90 (7.14)	51.27 (6.66)
Gender	24 female (48.98%)	9 female (60%)	1 female (10%)	8 female (72.73%)
Intellectual disability level at time of CAMDEX-DS interview	24 (49%) mild, 24 (49%) moderate, 1 (2%) unknown	2 (13%) mild, 12 (80%) moderate, 1 (7%) unknown	4 (40%) mild, 6 (60%) moderate	3 (27%) mild, 6 (55%) moderate, 2 (18%) unknown
CAMDEX-DS total score, mean (s.d.)	1.14 (2.40)	7.40 (7.87)	11.50 (6.50)	41.82 (23.61)
CAMDEX-DS Section A score, mean (s.d.)	0.10 (0.37)	1.20 (1.74)	1.70 (2.45)	7.82 (4.77)
CAMDEX-DS Section B score, mean (s.d.)	0.12 (0.39)	1.07 (1.71)	2.20 (1.62)	11.45 (6.28)
CAMDEX-DS Section C1 score, mean (s.d.)	0.49 (1.52)	2.27 (3.01)	4.10 (3.03)	14.00 (8.65)
CAMDEX-DS Section C2 score, mean (s.d.)	0.43 (0.79)	2.87 (2.85)	3.50 (3.47)	8.55 (7.51)

DSasymptomatic, asymptomatic; DSmentalhealth+, mental health condition but no Alzheimer's disease; DSprodromal, prodromal Alzheimer's disease; DSdementia, Alzheimer's disease.

### Correspondence of CAMDEX-DS scores to prodromal Alzheimer's disease and Alzheimer's disease dementia diagnoses

Kruskal–Wallis tests showed significant differences between diagnostic groups for CAMDEX-DS total score (*χ*^2^(3) = 56.191, *P* < 0.001, *ε*^2^ = 0.669), Section A score (*χ*^2^(3) = 40.844, *P* < 0.001, *ε*^2^ = 0.486), Section B score (*χ*^2^(3) = 54.627, *P* < 0.001, *ε*^2^ = 0.65), Section C1 score (*χ*^2^(3) = 47.09, *P* < 0.001, *ε*^2^ = 0.561) and Section C2 score (*χ*^2^(3) = 40.102, *P* < 0.001, *ε*^2^ = 0.477).

**Fig. 1:**
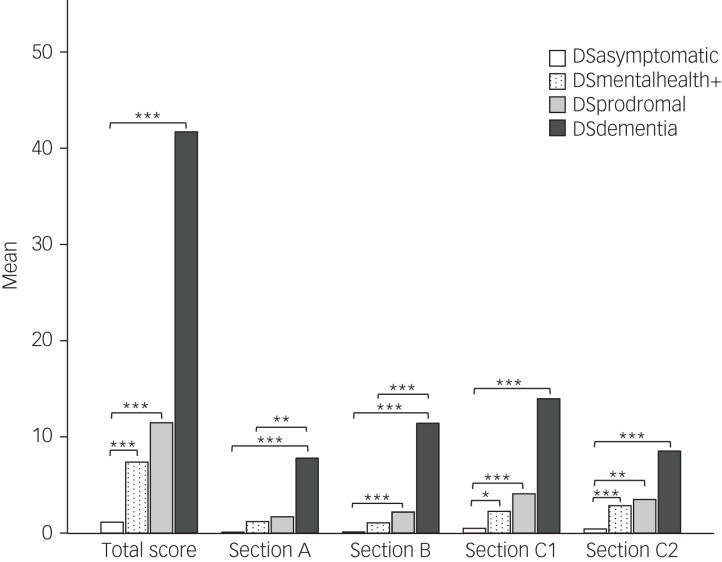
Mean score for each CAMDEX-DS section in each diagnostic group. Pairwise comparisons using Dunn's test with Bonferroni correction. *P ≤ 0.05, **P ≤ 0.01, ***P ≤ 0.001. CAMDEX-DS, Cambridge Examination for Mental Disorders of Older People with Down's Syndrome and Others with Intellectual Disabilities; DSasymptomatic, asymptomatic; DSdementia, Alzheimer's disease; DSmentalhealth+, mental health condition but no Alzheimer's disease; DSprodromal, prodromal dementia.

*Post-hoc* Dunn's tests with Bonferroni correction revealed that the DSmentalhealth+ group had significantly higher CAMDEX-DS total, Section C1 and Section C2 scores than the DSasymptomatic group (*P* = 0.001, *P* = 0.027 and *P* = 0.001, respectively). (Fig. 1).

DSdementia and DSprodromal groups had a significantly higher CAMDEX-DS total, Section B, Section C1 and Section C2 scores compared with the DSasymptomatic group (DSdementia: *P* < 0.001, *P* < 0.001, *P* < 0.001 and *P* < 0.001, respectively; DSprodromal: *P* < 0.001, *P* < 0.001, *P* = 0.001 and *P* = 0.005, respectively). CAMDEX-DS Section A scores were also significantly higher in the DSdementia group compared with the DSasymptomatic group (*P* < 0.001) (Fig. 1).

Significant differences were found between DSdementia and DSmentalhealth+ groups for Section A, Section B and Section C1 scores (*P* = 0.006, *P* < 0.001 and *P* = 0.010, respectively). No significant differences in CAMDEX-DS scores were found between DSprodromal and DSmentalhealth+ groups (total score, *P* = 1; Section A score, *P* = 1; Section B score, *P* = 0.167; Section C1 score, *P* = 1; Section C2 score, *P* = 1) (Fig. 1).

**Fig. 2:**
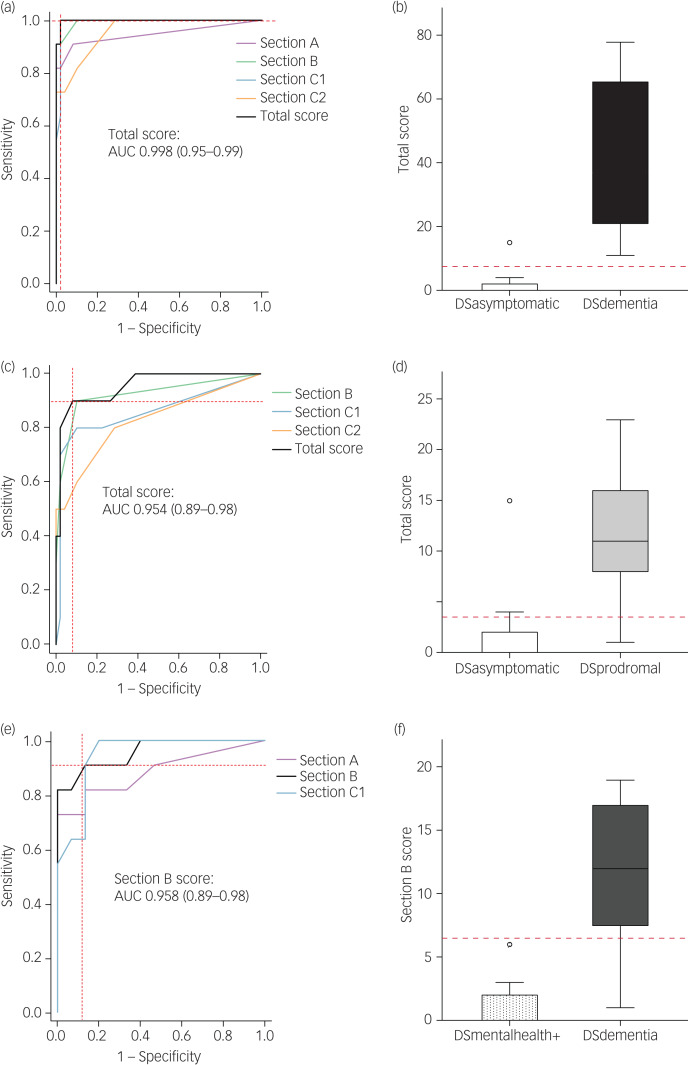
Correspondence of CAMDEX-DS scores and diagnosis. CAMDEX-DS total score was higher in DSdementia (b) and DSprodromal (d) participants compared with DSasymptomatic participants. CAMDEX-DS Section B score was higher in DSdementia participants compared with DSmentalhealth+ participants (f). Receiver operating characteristic analyses demonstrate a trend toward a higher AUC for prediction of diagnosis of DSdementia (a) and DSprodromal (c) when considering the CAMDEX-DS total score for participants in the DSasymptomatic, DSdementia and DSprodromal groups. Receiver operating characteristic analyses also show a higher AUC for prediction of DSdementia (e) diagnosis, when considering CAMDEX-DS Section B score for participants in the DSmentalhealth and DSdementia groups. The area under the curve is noted with 95% confidence intervals. Dashed red lines depict cut-off values based on the maximum Youden Index (a–f). AUC, area under the curve; CAMDEX-DS, Cambridge Examination for Mental Disorders of Older People with Down's Syndrome and Others with Intellectual Disabilities; DSasymptomatic, asymptomatic; DSdementia, Alzheimer's disease; DSmentalhealth+, mental health condition but no Alzheimer's disease; DSprodromal, prodromal dementia.

Where a significant difference was found, subsequent ROC analyses were carried out to assess the ability of CAMDEX-DS scores to classify between the DSdementia and DSprodromal groups (Fig. 2).

Including CAMDEX-DS scores for DSasymptomatic and DSdementia groups, ROC analyses demonstrated that CAMDEX-DS total, Section A, Section B, Section C1 and Section C2 scores were good classifiers of a diagnosis of Alzheimer's disease dementia, with AUCs of 0.998 (95% CI 0.953–0.999), 0.946 (95% CI 0.876–0.978), 0.994 (95% CI 0.946–0.999), 0.992 (95% CI 0.943–0.999) and 0.958 (95% CI 0.892–0.984), respectively. A CAMDEX-DS total score of >7.5 was considered positive and had the maximum Youden Index of 98 (Fig. 2, a and b).

Including CAMDEX-DS scores for the DSasymptomatic and DSprodromal groups, CAMDEX-DS total, Section B, Section C1 and Section C2 scores were also good classifiers of a diagnosis of prodromal Alzheimer's disease, with AUCs of 0.954 (95% CI 0.887–0.982), 0.923 (95% CI 0.846–0.963), 0.858 (95% CI 0.694–0.994) and 0.826 (95% CI 0.657–0.994), respectively. A CAMDEX-DS total score of >3.5 was considered positive and had the maximum Youden Index of 81.8 (Fig. 2, c and d).

Including CAMDEX-DS scores for DSmentalhealth+ and DSdementia groups, ROC analyses demonstrated that CAMDEX-DS Section A, Section B and Section C1 scores were good classifiers of a diagnosis of Alzheimer's disease dementia, with AUCs of 0.885 (95% CI 0.738–0.984), 0.958 (95% CI 0.892–0.984) and 0.954 (95% CI 0.887–0.982), respectively. A CAMDEX-DS Section B score of >6.5 was considered positive and had the maximum Youden Index of 81.8 (Fig. 2(e) and 2(f)).

Because no significant differences were found between participants’ CAMDEX-DS scores in the DSprodromal group compared with the DSmentalhealth+ group, ROC analysis including DSmentalhealth+ was not performed to establish a diagnosis of prodromal Alzheimer's disease.

### CAMDEX-DS scores and their relationship to Aβ binding and cortical thickness

A subcohort of 39 participants had data available from amyloid PET and structural MRI scans in addition to the CAMDEX-DS interview (for subcohort characteristics, see Supplementary Table 1). For all participants in this subcohort, the average interval between the CAMDEX-DS interview and scans was 0.82 ± 1.4 months, with a range of 0–5 months.

**Fig. 3:**
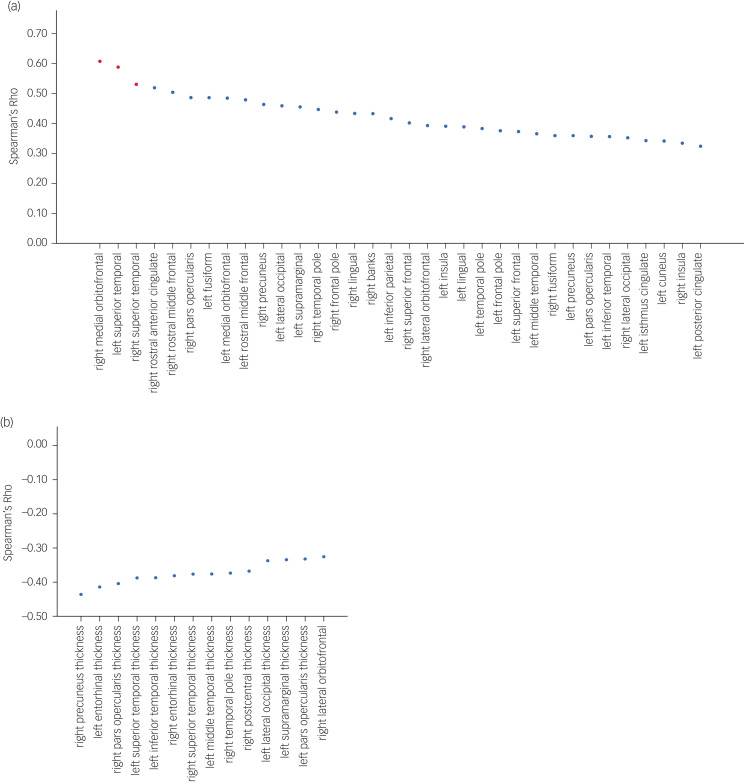
Regional correlation of Aβ binding and cortical thickness across the cortex and CAMDEX-DS total score. (a) Regions of Aβ binding with significant correlations plotted. Red data points indicate regions that remained significantly correlated after *P*-values were adjusted with the Bonferroni method. (b) Regions of cortical thickness with significant correlations plotted. No region remained significantly correlated after *P*-values were adjusted with the Bonferroni method. Aβ, amyloid β; CAMDEX-DS, Cambridge Examination for Mental Disorders of Older People with Down's Syndrome and Others with Intellectual Disabilities.

BP_ND_ and CAMDEX-DS total score were significantly correlated in 34 regions, with the peak correlations predominantly localised within frontotemporal regions (*r* ~ 0.4–0.6, *P* ≤ 0.05), after accounting for age and level of intellectual disability. Three of these regions (right medial orbitofrontal, left superior temporal and right superior temporal) survived the Bonferroni multiple comparison procedure at *P* ≤ 0.05 (Fig. 3a).

Cortical thickness and CAMDEX-DS total score were also significantly negatively corelated in 14 regions, with parietal, temporal and frontal regions exhibiting the strongest negative correlations (*r* ~ −0.4 to −0.44, *P* ≤ 0.05), after accounting for age and level of intellectual disability. However, none of these regions survived the Bonferroni multiple comparison procedure at *P* ≤ 0.05 (Fig. 3b); this was not surprising, given the conservative nature of this correction and the limited statistical power inherent in the sample size.

## Discussion

### Diagnostic thresholds to classify participants with Alzheimer's disease

To the best of our knowledge, this is the first study to derive diagnostic threshold scores for the CAMDEX-DS informant interview. Using ROC analyses to assess the accuracy of CAMDEX-DS scores to diagnose dementia against and the gold-standard psychiatric diagnosis, total scores greater than 7.5 and 3.5 were shown to classify participants into the Alzheimer's disease diagnosis group and prodromal Alzheimer's disease group, respectively, with a high degree of accuracy when compared with healthy adults with Down syndrome. A CAMDEX-DS memory and orientation score of ≥6.5 was able to classify participants into the Alzheimer's disease diagnosis group when compared with adults with Down syndrome and mental health conditions but no dementia; CAMDEX-DS scores were unable to accurately classify prodromal Alzheimer's disease when compared with adults with Down syndrome with mental health conditions but no dementia.

The thresholds derived here provide a standardised diagnostic process that allow for the use of the CAMDEX-DS in a research setting without the need for an experienced clinician to be present, reducing research costs. These thresholds also allow for more straightforward screening at a primary care level without the need for interviews at multiple time points.

### Association of CAMDEX-DS scores with Aβ binding and cortical thickness

The close mapping of CAMDEX-DS total scores with cerebral Aβ binding (as measured by Pittsburgh compound B binding to Aβ deposits) and cortical thickness further validates the utility of the derived scores, and demonstrates their ability to provide a clinical representation of *in vivo* pathological Alzheimer's disease markers in people with Down syndrome. The association between CAMDEX-DS total score and cortical atrophy in temporoparietal and frontal areas observed in this study is similar to recent reports, where atrophy in these brain regions has been associated with Alzheimer's disease in people with Down syndrome.^8^ Moreover, the predominance of temporal areas among the brain regions correlated with CAMDEX-DS total score here reflects previous studies, in which temporal lobe atrophy has been frequently reported in amyloid-positive people with Down syndrome.^9^ Additionally, the correspondence of CAMDEX-DS total score and Aβ binding was most evident in frontotemporal regions, consistent with previous studies tracking the amyloid pathology related to dementia diagnostic status in adults with Down syndrome.^6,10^ Keator et al^11^ identified that Aβ binding particularly in the middle and superior orbitofrontal and the superior temporal lobes were associated with dementia status, mirroring our results showing strongest correlations with CAMDEX-DS total score in these regions. However, in a more recent study comparing mild cognitive impairment in people with Down syndrome (analogous to prodromal Alzheimer's disease in this study) with cognitively stable participants, Keator et al^12^ found only small differences in orbitofrontal Aβ binding. Given the inclusion of patients with Down syndrome and mild cognitive impairment, but not those with more advanced stages of Alzheimer's disease, Keator et al^12^ hypothesised that this weaker relationship may be because increases in orbitofrontal Aβ are an indicator of more advanced disease progression in Down syndrome. Taken together, it is possible that CAMDEX-DS scores effectively reflect later stages of Alzheimer's disease.

Although orbitofrontal Aβ binding exhibited the strongest correlations (i.e. later-stage Alzheimer's disease pathology), frontal and temporal regions were more generally correlated with CAMDEX-DS total score. Impairment of the frontal and temporal lobes, presented clinically in the form of emotional and behavioural difficulties and executive dysfunction, are thought to be implicated in the early stages of dementia in the Down syndrome population.^13,14^ The close mapping between CAMDEX-DS total score and Aβ binding in frontotemporal regions, coupled with the ability to accurately diagnose prodromal Alzheimer's disease in those without mental health conditions, suggests that CAMDEX-DS scores are, to some extent, reflecting these early changes.

### Limitations

#### Diagnosis of prodromal Alzheimer's disease

Of note, CAMDEX-DS scores between participants with Alzheimer's disease and those with mental health conditions without Alzheimer's disease differed for sections assessing everyday skills (Section A), memory and orientation (Section B) and other cognitive abilities (Section C1). However, scores did not differ for the section assessing personality, behaviour and self-care (Section C2). Given that Section C2 scores did differ between participants who were asymptomatic and those with Alzheimer's disease, this section may not effectively distinguish between behaviours caused by mental health conditions, and those caused by Alzheimer's disease. Thus, Section C2 scores may be effectively distorted when assessing those with mental health conditions compared with those without. A diagnosis of prodromal Alzheimer's disease from CAMDEX-DS scores is likely to be reliant on Section C2 because of the clinical presentation of frontotemporal lobe dysfunction. Indeed, the distortion of Section C2 scores partially explains why CAMDEX-DS scores lose their predictive power when distinguishing those with prodromal Alzheimer's disease from those with mental health conditions without dementia, and supports the notion that emotional and behavioural difficulties are indeed early signs of dementia in people with Down syndrome. Clinical input, referring back to the specified ICD-10, DSM-IV and CAMDEX-DS diagnostic guidance, during the early stages of dementia is still required. Although completing a CAMDEX-DS interview at a second time point may aid diagnosis, further work to increase the sensitivity of the CAMDEX-DS scores to the early signs of dementia in those with Down syndrome who also have mental health conditions is needed.

#### Thresholds across a range of intellectual disability and ages

All participants in this study had either a moderate or mild intellectual disability. It was therefore not possible to assess whether the thresholds derived in this study are accurate for those with a more severe intellectual disability. However, given that the CAMDEX-DS focuses on changes from a premorbid level of functioning, thus accounting for differences in baseline ability, it is likely that the level of intellectual disability has little effect on threshold scores. However, further assessment of thresholds across intellectual disabilities is needed.

Because of the lack of older people with Down syndrome in the groups without dementia in this study, the derived CAMDEX-DS diagnostic threshold scores may underrepresent the cognitive and functional features of older people with Down syndrome. However, since ageing in people with Down syndrome is invariably associated with Alzheimer's disease pathology, it may be difficult to disentangle the features of normal ageing in this group. Nevertheless, further studies including a cohort with a broader age range are needed to confirm these threshold scores across the Down syndrome lifespan.

#### Circularity of scores

A further potential limitation of this study concerns the possible circularity of scores, given that clinical diagnosis provided by the psychiatrist was derived from the CAMDEX-DS interview responses; the same responses used to generate a numerical CAMDEX-DS score. However, the aim of this study was to codify CAMDEX-DS, producing a numerical score to remove the need for specialist clinical knowledge rather than to validate the CAMDEX-DS (see Ball et al^4^ for validation of the CAMDEX-DS). At present, a diagnosis of dementia in people with Down syndrome ultimately relies on a psychiatrist's clinical judgement after reviewing an individual's clinical history. Here, the CAMDEX-DS interview details the structured clinical history, and the proposed threshold scores act as a proxy for the psychiatrist's judgement; the numerical scores effectively codify the specialist knowledge and clinical experience that psychiatrists apply to the CAMDEX-DS interview responses to arrive at a diagnosis. These threshold scores can be applied to the CAMDEX-DS interview, and a diagnosis achieved without a psychiatrist present.

To conclude, in clinical practice and research of dementia in people with Down syndrome, there is a need for a clear and concise, but broad-ranging, diagnostic tool. The CAMDEX-DS was designed for this purpose, and we present a valuable addition of diagnostic thresholds and scoring criteria. Although further research is needed to confirm these findings across a broader age range and levels of intellectual disability, the diagnostic threshold scores outlined in this study provide a basis for the accurate diagnosis of Alzheimer's disease dementia and prodromal Alzheimer's disease in adults with Down syndrome, without the need for specialist knowledge and/or lengthy and costly training. The characteristics of the CAMDEX-DS also allows for accurate cross-sectional diagnosis, thereby further reducing testing demands and costs.

## Data Availability

The data that support the findings of this study are available from the corresponding author, J.A.B-W., upon reasonable request.

## Supplementary material

For supplementary material accompanying this paper visit http://dx.doi.org/10.1192/bjo.2021.36. click here to view supplementary material
