# Supplementary material for: Establishing diagnostic thresholds for Alzheimer's disease in adults with Down syndrome: the Cambridge Examination for Mental Disorders of Older People with Down's Syndrome and Others with Intellectual Disabilities (CAMDEX-DS)
Source: BJPsych Open. 2021 Apr 13;7(3):e79. doi: 10.1192/bjo.2021.36 (PMC8086396; doi:10.1192/bjo.2021.36)
Supplement: Supplementary file 1 [file bjosup.zip › S2056472421000363sup001.docx]

**Supplementary Material 1.**

**Section A – Everyday Skills**

33. Does he/she have difficulties with his/her usual daytime activities at work, college or day centre?

34. Does he/she have difficulty with a special skill or hobby?

35. Does he/she have difficulty with shopping?

36. Does he/she have difficulty making a cup of tea?

37. Does he/she have difficulty with housework e.g. dusting, dishwashing?

38. Does he/she have difficulty preparing simple meals/snacks?

39. Does he/she have difficulty using the telephone?

**Section B – Memory And Orientation**

42. Does he/she have difficulty remembering recent events e.g. when he/she last saw you or what happened the day before?

43. Does he/she often have difficulty remembering where he/she has left things?

44. Does he/she have difficulty remembering what has been said and repeat the same question over and over?

45. Does he/she have difficulty in remembering short lists of items, e.g. shopping?

46. Does he/she have difficulty remembering significant events from his/her past?

47. Does he/she have difficulty remembering the names of close friends, relatives or carers?

48. Does he/she have difficulty in interpreting surroundings, e.g. knowing where he/she is?

49. Does he/she have difficulty finding the way around the neighbourhood, e.g. to the shops or Post Office near home?

50. Does he/she have difficulty finding the way around the home (or ward), e.g. finding the toilet?

51. Does he/she have difficulty knowing what day it is?

52. Does he/she have difficulty knowing what time of day it is?

**Section C1 – Other Cognitive Skills**

57. Does he/she find it difficult to keep his/her mind on things? Is he/she easily distracted?

58. Does his/her thinking seem slow?

59. Does his/her thinking seem muddled?

60. Does he/she have difficulty with reading?

61. Does he/she have difficulty in following instructions?

62. Does he/she have difficulty in keeping up with ordinary conversation?

63. Does he/she have difficulty with writing?

64. Does he/she speak very little?

65. When speaking, does he/she have difficulty in finding the right word or use wrong words?

66. Does he/she have difficulty identifying or recognising objects?

67. Does he/she have difficulty identifying or recognising people?

68. Does he/she have difficulty carrying out familiar complex tasks (such as getting dressed) despite the physical ability to do them?

69. Does he/she have difficulty in planning ahead and thinking about the future?

70. Does he/she have difficult to make decisions?

71. Does he or she have difficulty solving day-to-day problems?

**Section C2 – Personality, Behaviour And Self Care**

74. Does he/she behave in a manner that leads to social difficulties?

75. Would you describe him/her as lacking in personality?

76. Does he/she show little emotion? Would you describe him/her as emotionally flat?

77. Is he/she changeable in mood? i.e. Does he/she have rapid shifts between different emotions?

78. Does he/she show a lack of enthusiasm for his/her usual interests?

79. Is he/she often irritable or angry?

80. Does he/she show lack of concern for other people?

81. Does he/she act impulsively, by doing the first thing that comes to mind?

82. Is he/she stubborn or perhaps a little awkward?

83. Does he/she get involved in difficult or embarrassing situations in public because of his/her behaviour?

84. Does he/she engage in inappropriate sexual behaviour?

85. Is he/she very restless? For example does he/she find it hard to sit down for any length of time?

86. Does he/she repeat the same word or phrase over and over again?

87. Does he/she develop routines from which he/she cannot easily be discouraged?

88. Does he/she often try to eat far too much?

89. Does he/she try to eat peculiar things, such as soap, cigarettes or dirt?

93. Does he/she have difficulty feeding him/herself?

94. Does he/she have difficulty in dressing or undressing?

95. Does he/she have difficulty with grooming, e.g. combing hair, shaving?

96. Does he/she have difficulty with bathing or showering?

97. Does he/she wet or soil him/herself?
