# Supplementary material for: Establishing diagnostic thresholds for Alzheimer's disease in adults with Down syndrome: the Cambridge Examination for Mental Disorders of Older People with Down's Syndrome and Others with Intellectual Disabilities (CAMDEX-DS)
Source: BJPsych Open. 2021 Apr 13;7(3):e79. doi: 10.1192/bjo.2021.36 (PMC8086396; doi:10.1192/bjo.2021.36)
Supplement: Supplementary file 1 [file bjosup.zip › S2056472421000363sup002.pdf]

Participants with amyloid PET & MRI scan

	DSasymptomatic	DSmentalhealth+	DSprodromal	DSdementia
Number of participants	22	4	5	8
Age at CAMDEX interview, years, mean (s.d.)	39.18 (s.d. = 6.59)	31.25 (s.d. = 1.89)	47.60 (s.d. = 4.10)	49.88 (s.d. = 7.32)
Sex	9 female (40.91%)	2 female (50%)	0 female (0%)	6 female (75%)
ID level at time of CAMDEX-DS interview	10 (45%) mild, 12 (55%) moderate	1 (25%) mild, 3 (75%) moderate	2 (40%) mild, 3 (60%) moderate	3 (37%) mild, 4 (50%) moderate, 1 (13%) unknown
CAMDEX Total score, mean (s.d.)	0.68 (s.d. = 1.04)	13.25 (s.d. = 12.34)	13.06 (s.d. = 7.03)	45.50 (s.d. = 22.37)
CAMDEX Section A score, mean (s.d.)	0.14 (s.d. = 0.35)	2.50 (s.d. = 2.89)	2.20 (s.d. = 3.19)	7.88 (s.d. = 5.44)
CAMDEX Section B score, mean (s.d.)	0.09 (s.d. = 0.29)	2.75 (s.d. = 2.50)	2.60 (s.d. = 1.95)	11.50 (s.d = 6.61)
CAMDEX Section C1 score, mean (s.d.)	0.45 (s.d. = 1.14)	3.00 (s.d. = 3.56)	5.00 (s.d. = 3.16)	12.25 (s.d. = 9.02)
CAMDEX Section C2 score, mean (s.d.)	0.45 (s.d. = 1.06)	5.00 (s.d. = 4.16)	3.80 (s.d. = 2.28)	9.38 (s.d. = 8.85)
Global BP_{ND}, mean (s.d.)	18.45 (s.d. = 5.90)	18.45 (s.d. = 2.76)	29.94 (s.d. = 8.99)	35.65 (s.d. = 13.37)
Cortical thickness across all regions, mm, mean (s.d.)	2.74 (s.d. = 0.08)	2.67 (s.d. = 0.10)	2.59 (s.d. = 0.20)	2.65 (s.d. = 0.07)
